# Use of technologies in neurosurgery – a national survey of the section “techniques and innovations”

**DOI:** 10.1007/s10143-025-03955-7

**Published:** 2025-11-24

**Authors:** Fatemeh Khafaji, Christoph Sippl, Julian Prell, Stefan Linsler

**Affiliations:** 1 Klinik für Neurochirurgie, Klinikum Bayreuth, Medizincampus Oberfranken FAU Erlangen-Nürnberg, Hohe Warte 8, Bayreuth, 95455 Germany; 2 Klinik für Neurochirurgie, Universitätsmedizin Halle, Halle(Saale), 06120 Germany

**Keywords:** Techniques and innovations, New technology, Neurosurgery, Education

## Abstract

Advancements in neurosurgery have transformed the field, enabling safer, minimally invasive procedures supported by modern navigation, neuromonitoring, and intraoperative imaging techniques. The integration of robotics and navigation systems further enhances surgical precision, while advanced imaging modalities like intraoperative MRI and CT scans allow real-time visualization, facilitating more accurate tumor removal and hardware placement. Emerging technologies such as virtual reality and augmented reality are revolutionizing surgical planning, education, and patient-specific modeling. Despite these innovations, the application and education regarding these tools remain limited. This study aims to provide a comprehensive report on the availability of advanced techniques and facilities, as well as the quality of education imparted in Germany. To address this, a nationwide survey was conducted among 89 departments to evaluate the availability and utilization of modern neurosurgical technologies. The survey revealed that university hospitals tend to possess more advanced equipment, perform higher surgical volumes, and offer 24/7 services more frequently than other centers. Techniques such as intraoperative neuromonitoring, neuronavigation, and advanced imaging are widely available, particularly in academic centers; however, gaps exist in their consistent implementation across all hospitals. Residency programs increasingly incorporate training in these innovative techniques, with a strong correlation between university hospital status and educational opportunities. The survey highlights the need for broader dissemination of these technologies and standardized training to ensure all centers can provide high-quality, around-the-clock neurosurgical care. Overall, ongoing research, technological integration, and education are vital for advancing neurosurgical outcomes and expanding access to cutting-edge treatments across Germany.

## Introduction

Advancements in neurosurgery have revolutionized the field, offering patients safer and more effective treatment options for various neurological conditions. From minimally invasive procedures to cutting-edge technologies, neurosurgeons continually explore new techniques to enhance patient outcomes and improve quality of life [1–6].

One of the most significant advancements in neurosurgery is the development of minimally invasive techniques based on modern navigation systems, neuromonitoring, and intraoperative imaging methods. Intraoperative Neurophysiological Monitoring (IONM) plays a crucial role in ensuring the safety and efficacy of neurosurgical procedures [1, 6, 7]. IONM has demonstrated the ability to increase the extent of resection, improve postoperative neurological outcomes, and enhance survival rates significantly [8–10]. IONM involves the real-time monitoring and assessment of neurological function during surgery to prevent potential damage to the nervous system and optimize surgical outcomes. Thus, neurosurgery cannot possibly be imagined without it.

Furthermore, neuronavigational systems have revolutionized the neurosurgical process through precise tumor localization and detailed anatomical navigation, which immensely improve the overall resection rate and neurological outcomes [11, 12]. 

On the other hand, the integration of robotics has transformed neurosurgical practice. Robotics assist surgeons in performing precise, delicate procedures with enhanced accuracy and control [5, 13–15].

Another innovation in neurosurgery is using advanced imaging modalities, such as intraoperative MRI and intraoperative CT scanning [1, 16, 17]. These technologies allow surgeons to visualize the brain or spine in real-time during surgery, facilitating more accurate tumor resection, compensation for brain shifting, ensuring optimal placement of implants or hardware, and improving the survival rate [18, 19] Nowadays, virtual reality (VR) and augmented reality (AR) are revolutionizing the field of neurosurgery, offering innovative tools and techniques to improve surgical planning, education, and patient outcomes. VR-based educational model proved to be useful for both inexperienced and experienced groups. Furthermore, both students and residents’ groups gave their positive feedback about learning using the VR and AR [20]. These immersive technologies provide neurosurgeons with new ways to visualize complex anatomical structures and simulate surgical procedures in a virtual environment. In surgical planning, VR and AR allow neurosurgeons to reconstruct patient-specific 3D models of the brain or spine using advanced imaging techniques such as MRI and CT scans.

Additionally, 3D printed models are more common [21, 22]. Overall, these models provide detailed anatomical information, enabling surgeons to preoperatively visualize the surgical site from any angle, thereby enhancing their understanding of the patient’s anatomy and pathology. The impact of augmented reality and virtual reality in the education of trainees should also be taken into account [23]. 

Overall, neurosurgery continues to evolve rapidly with the introduction of new techniques and methods aimed at improving patient outcomes and advancing our understanding of the brain and nervous system. Through ongoing research and innovation, neurosurgeons are at the forefront of medical progress, offering hope and healing to patients with complex neurological conditions.

However, the knowledge about the use of these technical tools, the application of new devices, and education in the application of technologies in the neurosurgical theatre in neurosurgery is low. Therefore, the section on techniques and innovations of the German Society of Neurosurgery performed a national survey to analyze the daily routine and the application of all these modern technologies in neurosurgery to draw the knowledge gap.

## Materials and methods

A 28-question survey was developed (see supplementary table). The type of questions asked was discrete (e.g., yes/no) and quantitative (e.g., number of microscopes).

The questionnaire was submitted to the chairmen of all neurosurgical departments of Germany by the German Society of Neurosurgery in August 2022.

The departments varied across university hospitals, maximum care/tertiary care providers, teaching hospitals, and municipal hospitals, as well as affiliated departments. The survey was conducted between August 2022 and October 2022.

Questions were sought to describe participants’ institutions, the detailed use of various techniques, their daily use, emergency use, education, and the application of new innovative methods. Responder rate calculations were not possible given the widespread dissemination of the survey. Nearly every department and outpatient clinic was represented.

The analysis and visualization of data were conducted using IBM^®^ SPSS^®^ Statistics Version 26 (SPSS Inc., Chicago, USA). Survey cohorts were compared using the Whitney-U-Test, Fisher’s exact test, and multivariate analysis to assess differences between values in the groups. The significance level was set at *p* < 0.05. The figures were created using GraphPad Prism Software Version 10.04.2.

## Results

### Hospital profiles and surgical volumes

The survey encompassed a diverse range of healthcare facilities, including 58 university hospitals and maximum care providers, alongside 11 dedicated teaching hospitals. Additionally, 18 municipal hospitals and affiliated departments contributed to the comprehensive dataset. Overall, a total of 89 responses (78%).

**Fig. 1:**
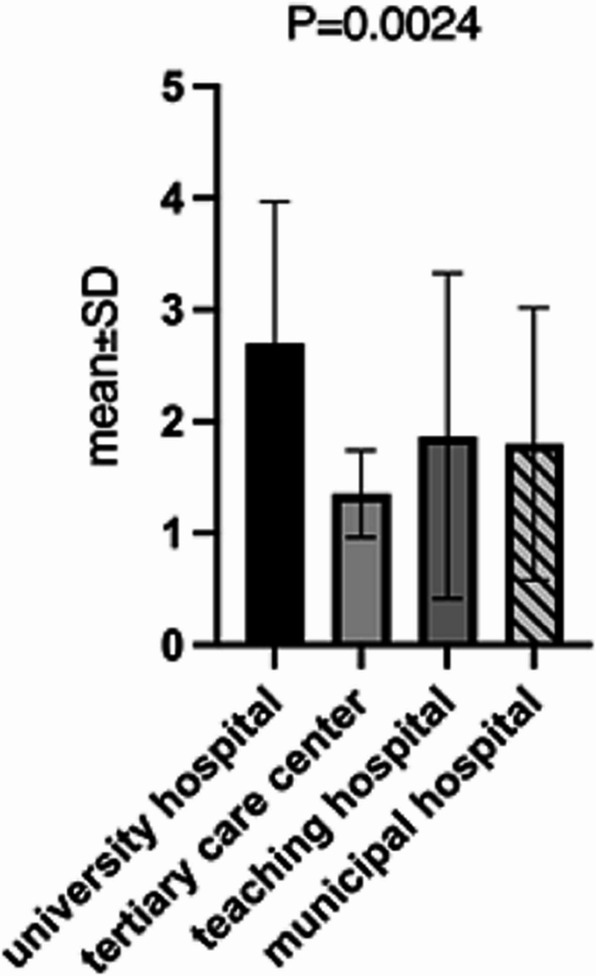
Capacity of surgical theatres (mean and SD) in correlation to hospital type

The number of surgeries performed annually at these institutions ranged from 500 to 5,000. The average number of available operating rooms was 2.35 ± 1.30 (minimum: 1, maximum: 6), with significantly higher availability in university hospitals (*p* = 0.001). On average, these institutions conducted 1,640.68 ± 1,060.23 surgeries per year, with university hospitals performing significantly more surgeries (t-test, *p* < 0.05). Details are illustrated in Fig. 1.

### Equipment availability

**Fig. 2:**
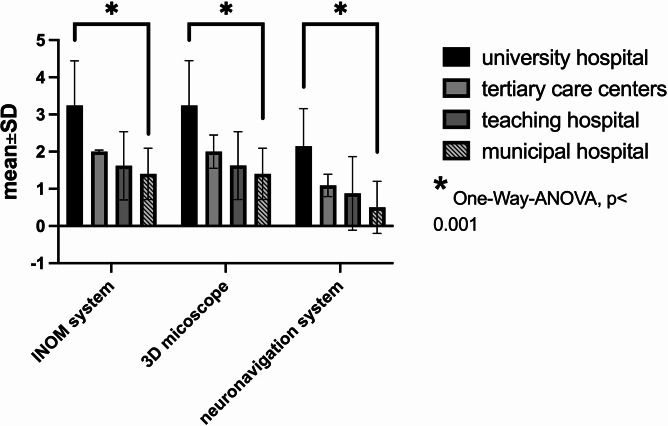
Number of neuromonitoring, microscopes and neuronavigation devices in the department

**Fig. 3:**
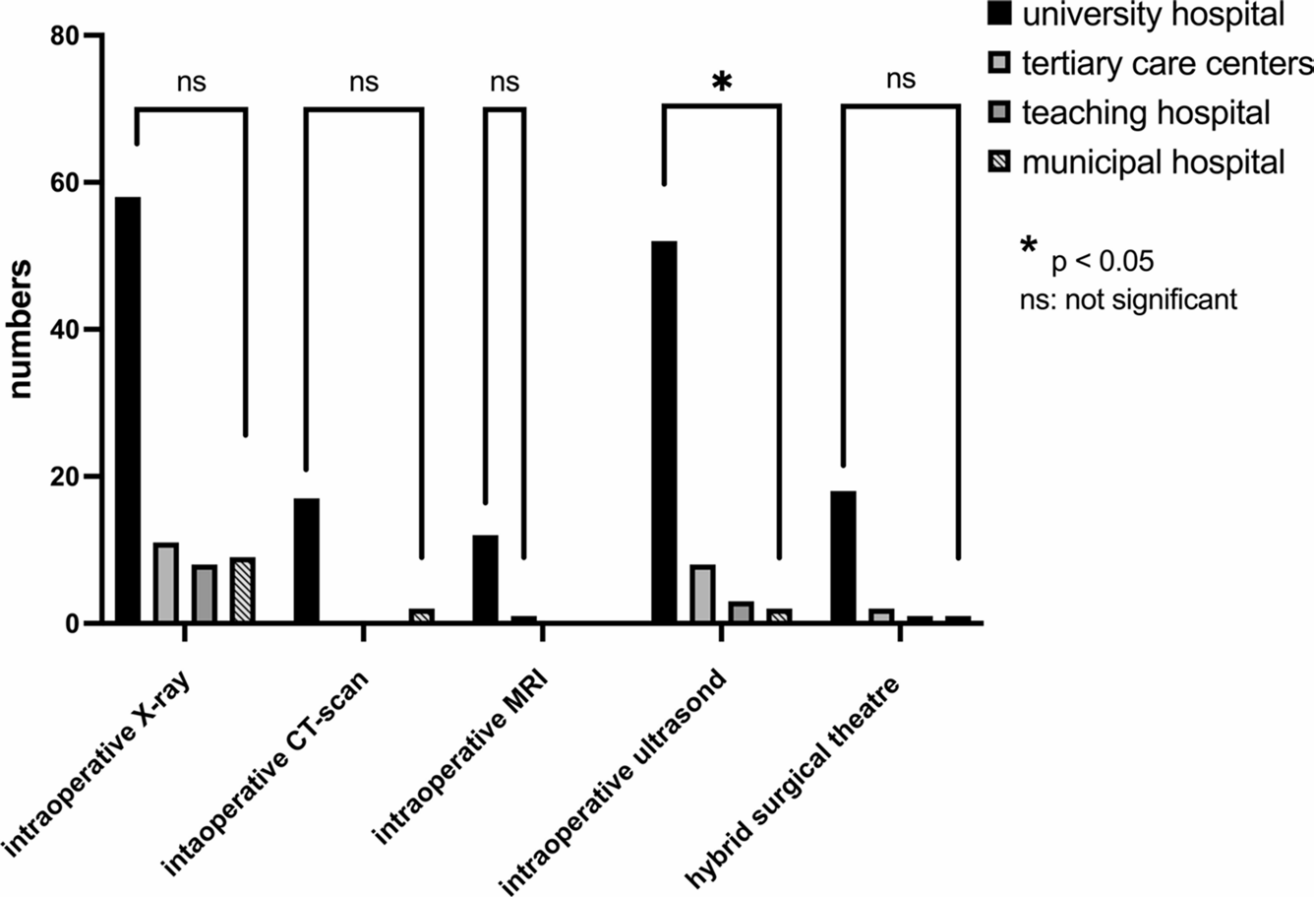
Number of departments with intraoperative X ray, CT, MRI, ultrasound and hybrid surgical theatre. There was no significance between the different hospital facilities beside availability of intraoperative ultrasound

**Fig. 4:**
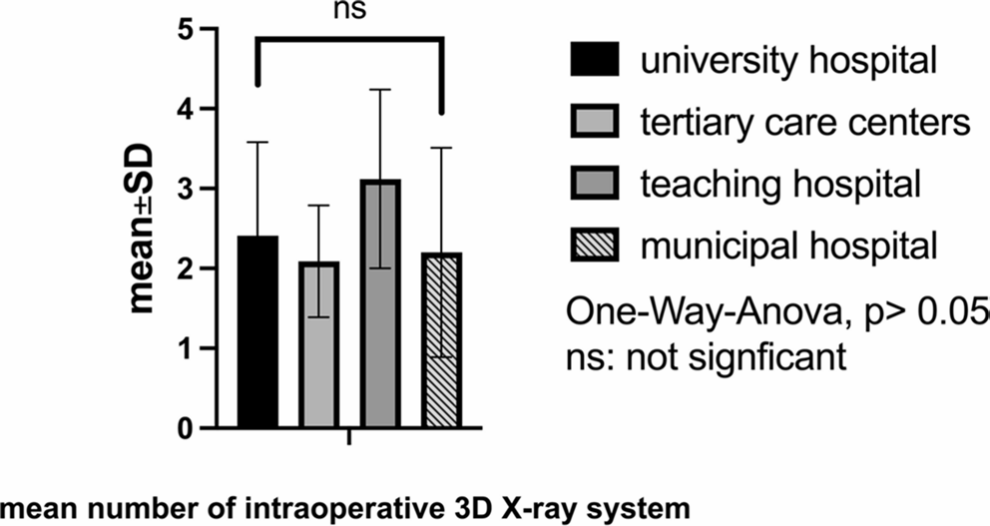
Mean number of intraoperative 3D x rays in the different hospitals facilities

The average number of operating room microscopes per department was 2.72 ± 1.29 (minimum: 1, maximum: 7, median: 3, *P* < 0.05). Departments also had an average of 1.7 ± 1.1 navigation systems (maximum: 5, median: 2, *P* < 0.05) and 2.41 ± 1.14 X-ray arcs (maximum: 6, median: 2, *P* > 0.05). Additionally, 73.6% (*n* = 64) of departments were equipped with 3D-scan X-rays or O-arm systems (median: 1). Intraoperative CT scans were available in 19 centers (21.8%), and only 13 centers (14.9%) had intraoperative MRI devices. Twenty-six centers (29.9%) had the capability for intraoperative angiography, and 22 centers (25.3%) offered hybrid surgeries. Details are illustrated in Figs. 2, 3 and 4.

Intraoperative ultrasound and Doppler devices were available in 74.4% of departments, with significantly higher availability in university hospitals (*p* < 0.001). Endoscopic systems for intraventricular, endonasal, and spinal procedures were available in 72, 60, and 40 centers, respectively. While spinal endoscopy showed no significant difference across departments, endonasal endoscopic procedures were significantly more common in university hospitals (*p* < 0.0001). Moreover, 33 departments utilized 3D microscopic systems, and 14 employed endoscopic systems.

Fluorescence-guided techniques were utilized in 24 centers, with 5-ALA applied in 64 centers (73.6%) and ICG-angiography available in 59 centers (67.8%).

Concerning new innovative techniques, the survey revealed that 3D microscopy is available in 38% of the departments, 3D endoscopic devices are available in 16% of the departments, and augmented reality is used in 21% of all departments nowadays. All these innovative techniques with significantly higher availability in university hospitals (*p* < 0.001).

### Intraoperative neuromonitoring and preoperative planning

Routine intraoperative neuromonitoring was available in 92% of centers, with an average of 1.44 ± 0.92 machines per operating room, predominantly in university hospitals (*p* < 0.0001). Comprehensive neuromonitoring modules were also more frequently utilized in university hospitals (*p* < 0.0001). Intraoperative EEG was available in 35.6% of departments, and awake craniotomies were performed in 53 centers (60%).

For preoperative planning of cranial surgeries, navigated transcranial magnetic stimulation (nTMS) is used in 26 neurosurgical departments, primarily in university hospitals (*p* = 0.007). Functional MRI and DTI-based fiber tracking were available in 37 and 58 departments, respectively, with significantly higher prevalence in university hospitals (*p* < 0.000) as well.

### 24-Hour service availability of technique and staff

Around-the-clock availability of complex spinal surgeries was reported by 81.6% of clinics (Spearman’s rank, *p* > 0.05). However, 24-hour cerebrovascular surgical services were offered by 61 departments (68%), predominantly in university hospitals (Spearman’s rank, *p* < 0.0001). Intraventricular endoscopic interventions and endonasal endoscopic surgeries were also more commonly available 24/7 in university hospitals (*p* < 0.0001 and *p* = 0.016, respectively). Only 19 departments (predominantly university hospitals) offered 24-hour neuromonitoring services as part of their daily routine (*p* < 0.0001). Spinal and cranial neuronavigation were routinely used 24/7 in 84 departments (95%). Details are demonstrated in Table 1.

**Table 1:** Around-clock availability of the different techniques and personal for neurosurgical emergency procedures

24/7availability	Neuronavigation	IONM	Vascular neurosurgery	Intraventricular neuroendoscopy	Endonasal neuroendoscopy	Complex spine surgery
university hospital and maximum care hospital	100%	38%	84%	62%	34%	84%
teaching hospital	100%	27%	72%	45%	36%	72%
municipal hospital	100%	0%	50%	0%	0%	88%
affiliated department	100%	0%	0%	0%	0%	70%

### Residency training and education in innovative techniques

**Fig. 5:**
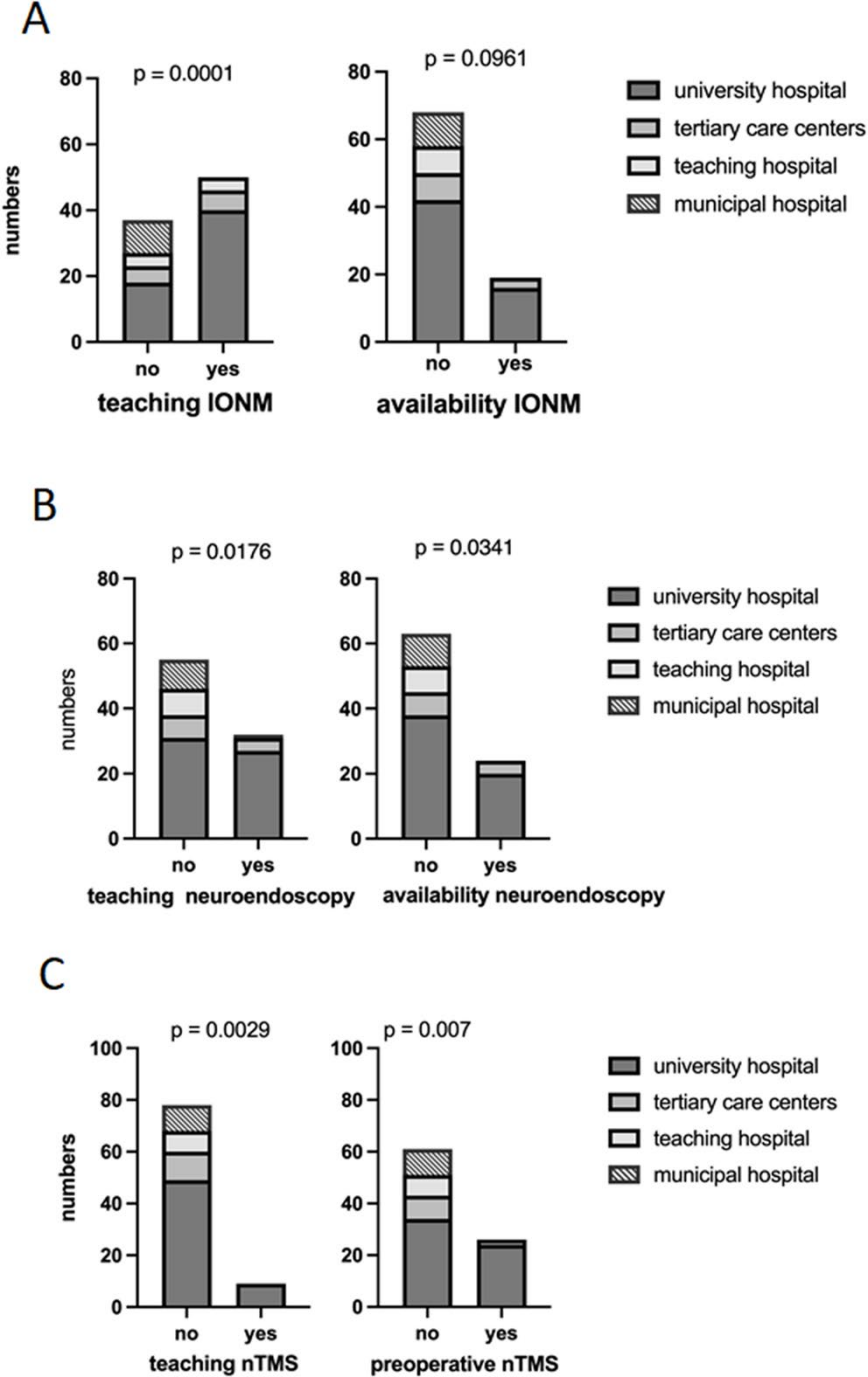
Correlation of availability and teaching in the different hospital facilities for IONM (**A**), neuroendoscopy (**B**) and nTMS (**C**)

Residency training programs incorporated instruction in intraoperative neuromonitoring in 57% of clinics, predominantly in university hospitals (*p* = 0.0001). Teaching nTMS was limited to nine university hospitals (10%), (*p* = 0.029). Neuroendoscopic training was available in 32 departments (36%), again primarily in university hospitals (*p* < 0.005) (see Table 2). Pearson correlation analysis revealed high significant correlation of hospital university to education in all devices and innovative techniques as well as number of cases and use of preoperative imaging and planning tools, e.g. fiber tracking and nTMS. Although, the 24/7availability did not correlate to the type of hospital. Correlation of availability and education is illustrated in Fig. 5.

**Table 2:** Capacity of surgical theatres (mean and SD) in correlation to hospital type

Education	Neuronavigation	IONM	nTMS	Neuroendoscopy
university hospital and maximum care hospital	100%	68%	15%	46%
teaching hospital	100%	54%	0%	0%
municipal hospital	100%	50%	0%	22%
affiliated department	0%	0%	0%	10%

## Discussion

The presented survey, conducted across various healthcare institutions in Germany, provides valuable insights into the landscape of medical facilities and technological capabilities. With a notable response rate of 78%, the data sheds light on current trends in healthcare provision and the adoption of cutting-edge technologies in neurosurgery. The survey focuses on two main aspects: the distribution of intraoperative equipment across neurosurgical departments and its daily use. Additionally, it examines how this infrastructure influences education and training. Thereby, the educational element was asked from the chair’s point of view.

The German Neurosurgical Society boasts one of the largest neurosurgical communities in Europe and has the highest number of neurosurgical residents. However, despite this strong foundation, only 5% of trainees report being satisfied with their surgical progress and mentorship [24]. While some studies have explored the relationship between neurosurgical equipment availability and the quality of educational programs, this area remains under-explored [2, 25, 26]. With over 140 hospitals recognized as neurosurgery training centers, a crucial question arises: How much training in new techniques and innovative procedures is routinely integrated into residents’ education? This survey represents the first comprehensive effort to assess the availability of neurosurgical equipment across departments in Germany.

Furthermore, advanced intraoperative infrastructure should empower neurosurgical departments to perform high-tech surgeries and deliver optimal treatment around the clock. Yet, questions remain: How many departments are equipped, both technically and personnel-wise, to consistently provide such services? More importantly, how many trainees gain independent experience with modern technical tools, enabling them to develop the skills essential for a successful career?

### Neurosurgical department infrastructure

German neurosurgical infrastructure expanded from 146 to 201 departments between 1997 and 2008, mainly due to private new hospitals [27]. The neurosurgeon workforce grew by 151% from 2000 to 2019, reaching 2.446 board-certified neurosurgeons, the highest density in Europe at 2.94 per 100,000 inhabitants. However, this growth wasn’t matched by increases in departments or procedures [28]. Rural neurosurgery faces challenges like long transport, resource shortages, and retaining specialists, with telemedicine and mobile teams as potential solutions [29]. University hospitals demonstrate significantly better intraoperative infrastructure and have more operating theaters. They are also more likely to introduce innovative techniques in neurosurgery. Naturally, this results in a higher volume of surgical procedures and greater surgical achievements.

While intraoperative neuromonitoring is generally a routine part of neurosurgical procedures, techniques such as navigated transcranial magnetic stimulation (nTMS), functional MRI (fMRI), and fiber tracking are primarily used for preoperative imaging, especially in university hospitals and maximum-care providers. Intraoperative imaging with MRI, CT, or hybrid operating rooms is available in only a minority of departments. Interestingly, intraoperative ultrasound is used in most neurosurgical departments. Notably, awake surgeries are performed in 60% of the responding hospitals, reflecting a growing trend toward patient-centered surgical approaches.

Neuroendoscopy is mainly employed for intraventricular surgeries. The use of endoscopy in endonasal skull base surgery and minimally invasive spinal procedures is common across about two-thirds of departments. Endonasal skull base surgery is more prevalent in university hospitals and maximum-care centers, while endoscopic spinal surgery is more common in teaching and municipal hospitals, although this difference is not statistically significant.

Non-university hospitals underuse new neurosurgical techniques due to barriers. Weber et al. found university hospitals scored higher on new technology use, with regional differences across Europe [30]. Economic constraints are key, as Ahmed et al. have shown that high equipment costs hinder the adoption of minimally invasive methods, with private hospitals being more likely to adopt them due to better resources [31]. Training issues also play a role, with a lack of training programs and often a lack of confidence in available facilities [32].

### 24/7 availability of major surgeries and techniques

Although university hospitals and maximum-care centers possess more advanced intraoperative equipment, our data reveal no significant difference in the availability of emergency spinal and neurovascular surgeries during night shifts. This points to a potential gap in utilizing existing infrastructure to ensure round-the-clock service.

Only 68% of all departments provide a 24-hour neurovascular surgical service, and even fewer can offer 24/7 emergency neuroendoscopic procedures. Shockingly, only 21% of departments provide continuous neuromonitoring, despite its importance in improving surgical outcomes. Given the critical role of neuromonitoring in enhancing patient safety and postoperative results, the authors urge the neurosurgical society to prioritize strengthening neurosurgical services nationwide. Neurosurgical care should be accessible 24/7 for every patient.

### Educational aspects

The relationship between theoretical and practical education during neurosurgical training varies across European countries [33]. The educational technique has undergone significant change since the pandemic era [34]. New didactic methods have been developed and refined. The ongoing advancements in VR and AR educational techniques and models suggest promising prospects for the academic education of residents [23].

Currently, given the limited educational resources available for trainees, the AR-based method has demonstrated the potential to revolutionize the traditional educational offering, innovative tools and techniques to improve surgical planning, education, and patient outcomes. Some institutions, such as the Barrow Institute, are pioneering efforts in this domain [23]. The recent studies are mainly helpful to learn 3D cerebral anatomy, which might help in the intraoperative orientation [35–37].

Limited research has been conducted on the influence of advanced intraoperative infrastructure on resident education, and information concerning training in novel neurosurgical techniques remains limited. Each cohort of residents encounters the challenge of acquiring proficiency in emerging technologies. As surgical procedures become increasingly intricate and shift towards minimally invasive methods, it is imperative to incorporate new technologies such as nTMS, intraoperative neurophysiological monitoring (IONM), and neuroendoscopy into educational curricula.

Integrating emerging technologies in neurosurgical education faces challenges despite its benefits. Virtual and augmented reality shows promise, with 61% of trainees agreeing it could become standard and 60% seeing it as cost-effective. Major barriers include limited training time (63%) and institutional access, with only 59% having extended reality access [38].

The adoption of surgical technologies follows a “follow the leader” mentality, where timing and familiarity are crucial factors beyond product quality [39].

Our survey highlights a gap between the availability of these new technologies and their integration into neurosurgical education. Developing a comprehensive curriculum that includes these innovations could improve both 24/7 service availability and the overall quality of neurosurgical care. Strengthening the commitment to incorporate modern technologies and concepts into training will ensure neurosurgery remains at the forefront of medical innovation.

This survey is among the first to explore the actual usage of techniques, their routine availability, and their impact on emergency cases and education. However, it has limitations: response bias is possible, as university hospitals — more closely linked to the German Society of Neurosurgery — were more likely to participate. Additionally, the questionnaire did not assess residents’ confidence in independently using equipment during night shifts when supervision is limited.

Interestingly, despite access to advanced intraoperative tools, only 8% of residents disagreed that such technology provides valuable learning experiences, and just 12% disagreed that intraoperative technology enhances productivity—findings consistent with a small US-based survey [1].

A significant limitation of our research is that the survey was exclusively completed by chairpersons, which complicates the interpretation of trainees’ satisfaction and confidence levels concerning their training, whether at the university hospital or a tertiary hospital. Therefore, it is recommended that a new survey be conducted specifically among neurosurgical trainees to address these concerns.

Finally, global neurosurgical training and infrastructure exhibit considerable disparities between high-income countries, as e.g. European countries, U.S. and U.K., and low- and middle-income countries, as e.g. Pakistan and India. Young neurosurgeons in high-income counties encounter fewer obstacles, with merely 12.5% citing limited availability of trained neurosurgeons as a barrier to service delivery, in contrast to 69.2% in low and middle-income countries [40].

## Conclusion

This survey offers a comprehensive overview of daily practices and the use of intraoperative and preoperative techniques in German neurosurgery, many of which have evolved over recent decades. The findings should influence policies around education and 24/7 service availability, ultimately aiming to provide every patient with the best possible neurosurgical care around the clock.

## Data Availability

No datasets were generated or analysed during the current study.

## Abbreviations

AR: augmented reality
cCT: cranial computer tomography
IONM: Intraoperative Neurophysiological Monitoring
MRI: magnetic resonance imaging
fMRI: functional magnetic resonance imaging
DTI: diffusion tensor imaging
nTMS: navigated transcranial magnetic stimulation
VR: virtual reality
